# LONG TERM IMPACT OF COVID-19 ON MENTAL HEALTH: A TUNISIAN STUDY

**DOI:** 10.1192/j.eurpsy.2023.1695

**Published:** 2023-07-19

**Authors:** N. Bouattour, M. Turki, W. Bouaziz, M. Abdelkefi, R. Jbir, S. Ellouze, S. Msaad, S. Kammoun, N. Halouani, J. Aloulou

**Affiliations:** 1 Psychiatry B; 2 Pneumology, Hedi Chaker University Hospital, Sfax, Tunisia

## Abstract

**Introduction:**

Patients with long COVID experience a wide range of physical and psychological symptoms. Mental health disturbances include cognitive impairment, memory loss, anxiety, depression sleep disorders…

**Objectives:**

We aimed to determine to assess mental repercussions in long COVID, especially sleep disturbances and depression.

**Methods:**

This is a prospective cohort study including 84 adults Tunisian COVID 19 inpatients who had been discharged alive from hospital. Each enrolled patient was asked about the period before SARS COV2 related hospital stay, and the 6-9 month-period after hospital discharge, using the validated Arabic version of *the Patient Health Questionnaire (PHQ)-9 and* the Insomnia Severity Index (ISI).

**Results:**

The mean age of patients was 57,59 ± 12,84 years with a sex ratio (H/F) 1,2.

As compared with baseline, all assessed outcomes (ISI and PHQ) significantly impaired after the covid-19 infection (p<0.001 for the two cases).

The prevalence of depressive symptoms doubled after the infection (25% to 58,3%).

The prevalence of insomnia was multiplied by 5 after the covid-19 infection (5,95% to 30,95%). ISI score was correlated with the PHQ score (p<0.001; r=0.738).

**Conclusions:**

Our study highlighted the association between COVID-19 infection and the impairment of mental health outcomes. Thus, patients who have experienced COVID-19 illness should be screened for long psychological disturbances even a few months after the infection, in order to guarantee a better quality of life.

**Disclosure of Interest:**

None Declared

